# Sora (*Porzana carolina*) autumn migration habitat use

**DOI:** 10.1098/rsos.171664

**Published:** 2018-05-16

**Authors:** Auriel M. V. Fournier, Doreen C. Mengel, David G. Krementz

**Affiliations:** 1 Arkansas Cooperative Fish and Wildlife Research Unit, Department of Biological Sciences, University of Arkansas, Fayetteville, AR, USA; 2 US Geological Survey, Arkansas Cooperative Fish and Wildlife Research Unit, Department of Biological Sciences, University of Arkansas, Fayetteville, AR, USA; 3 Resource Science Division, Missouri Department of Conservation, Columbia, MO, USA

**Keywords:** resource use, sora, *Porzana carolina*, autumn migration, wetland

## Abstract

Palustrine wetland management across the USA is often conducted under a moist soil management framework aimed at providing energetic resources for non-breeding waterfowl. Moist soil management techniques typically include seasonal water-level manipulations and mechanical soil disturbance to create conditions conducive to germination and growth of early successional, seed-producing wetland plants. The assumption is that providing stopover and wintering habitat for non-breeding waterfowl will also accommodate life-history needs of a broader suite of migratory waterbirds including shorebirds, wading birds and marsh birds. Although studies of wetlands provide some evidence to support this assumption for shorebirds and wading birds, there is less information on how other marshbirds respond. Sora (*Porzana carolina*) are a species of migratory rail that depend on wetlands year round as they migrate across North America. It is a species for which the consequences of wetland management decisions directed towards non-breeding waterfowl are unknown. We conducted nocturnal surveys on 10 public properties in Missouri, USA during autumn migration during 2012–2016 to examine sora habitat use in wetland impoundments managed to enhance the production of moist soil vegetation. We found a positive relationship with sora presence and mean water depth and annual moist soil vegetation; sora used, on average, deeper water than was available across surveyed impoundments and used locations with a higher percentage of annual moist soil vegetation than was available. We found a negative relationship with sora use and upland vegetation, woody vegetation and open water. We found sora using deeper water than have previously been reported for autumn migration, and that moist soil management techniques used on Missouri's intensively managed public wetland areas may be compatible with sora autumn migration stopover habitat requirements.

## 1. Introduction

Wetlands are among the nation's most imperiled ecosystems with the conterminous USA experiencing an estimated 53% wetland loss since pre-European settlement [1]. Freshwater emergent wetlands (wetlands dominated by erect, rooted, herbaceous hydrophytes, [2]) have suffered the highest per cent loss among all freshwater vegetated wetland types (i.e. forest, shrub and emergent) with approximately 17% of emergent wetlands remaining in 1954 lost in the 55-year period 1954–2009 [1]. The central US landscape is highly altered owing to agricultural conversion and associated infrastructure (i.e. levees and ditches), navigation considerations associated with major riverways and other human developments such as roads and urban expansion. The degree of altered processes (i.e. disrupted flood pulses, stream or river channels constrained by levees, ditching to decrease flood duration that once created and maintained riverine wetlands) requires that public rehabilitated wetlands be intensively managed to provide a variety of habitats and resources for wetland-dependent wildlife as well as to accommodate wetland recreational opportunities for the agency's stakeholders. A common management approach taken by wetland managers is to maximize production of early successional wetland plants; this approach is known as moist soil management [3]. Moist soil management is typically associated with providing energetic resources for autumn migrating waterfowl by creating conditions favourable to germination and growth of seed-producing plants adapted to moist or saturated growing conditions. The energetic resources (i.e. seeds) are made available by incrementally inundating wetland impoundments throughout late summer, autumn and early winter to provide habitat for non-breeding waterfowl, beginning with early migrating dabbling ducks (blue-winged teal, *Anas discors*) and continuing through to late-arriving dabbling ducks (i.e. mallards, *Anas platyrhynchos*).

A key assumption of moist soil wetland management is that providing habitat for the full suite of waterfowl species (Anatidae—ducks, geese and swans) will also accommodate life-history needs of a broader suite of waterbirds including shorebirds, wading birds and marshbirds. Although studies of moist soil wetlands provide some evidence to support this assumption regarding shorebirds and wading birds [4], there is less information on how marshbirds respond to the vegetative and habitat conditions resulting when wetlands are managed with a focus on non-breeding waterfowl. Marshbirds encompass a group of wetland-dependent birds including rails, bitterns and moorhens [4] that are generally inconspicuous and tend to inhabit wetlands with robust, perennial vegetation, making them difficult to detect [5]. Overall, limited distributions combined with low detection probability have resulted in marshbirds being among the least studied avian groups [6] and, for most species of marshbirds, there is limited information on population levels, breeding ecology, migration patterns and overall habitat requirements [7].

Our work focuses on one marshbird, the sora (*Porzana carolina*), a species of migratory rail that is a common autumn migrant and is often detected in wetland impoundments managed under a moist soil management scenario [8]. Sora are habitat generalists that forage primarily on seeds throughout autumn migration, which may explain why sora are found in high densities in thick moist soil vegetation in the autumn [9]. However, wetland management decisions, specifically timing of water-level manipulations, directed towards non-breeding waterfowl have unknown consequences on the suitability of stopover habitat for this species.

Much of the previous sora autumn migration research has been conducted in Missouri [8], although this previous work was completed on single sites and was based on opportunistic observations. Findings from these studies tend to indicate that sora use habitat based more on water conditions and plant structure rather than specific plant species [9–12]. Sora were detected during autumn migration in shallowly inundated (2–45 cm) habitats dominated by annual, seed-producing wetland vegetation, including *Panicum*, *Echinochloa* and *Bidens* spp. [10–12]. Sora have been found in high densities around wetlands that were dewatered (less than 15 cm) in accordance with local irrigation practices during late summer in Colorado, thereby reducing available habitat and concentrating food resources [13]. High densities of sora were also recorded on wetlands inundated (5–15 cm) during the early part of autumn migration (early September) in Missouri [11]. Sora migration in Missouri begins in early August and continues through to late October, with the peak of migration occurring in late September [14]. Our objective was to examine sora habitat use on public wetlands in Missouri during the entire span of their autumn migration under a standardized protocol to help inform wetland managers of the habitat use of sora during autumn migration, and to form a foundation for future work examining wetland management for the full suite of wetland bird species.

## 2. Material and methods

### 2.1. Study site

Missouri is one of six mid-latitude states in the USA (Iowa, Illinois, Indiana, Kentucky, Missouri and Ohio) that suffered greater than 80% loss of their historical wetland acres [1]. To compensate for this degree of wetland loss, the Missouri Department of Conservation (MDC) and the US Fish and Wildlife Service (USFWS) invested in acquiring and restoring wetland areas distributed geographically across Missouri. These Conservation Areas (public land parcels owned by MDC) and National Wildlife Refuges (public land parcels owned by USFWS) are located within riverine floodplains; seven are associated with mid-size rivers (stream order 6–8) and eight are associated with either the Missouri or Mississippi Rivers. These state and federal properties have served as core areas for the development of regional wetland complexes as wetland development on private land has occurred in close juxtaposition to the public properties.

**Figure 1.**
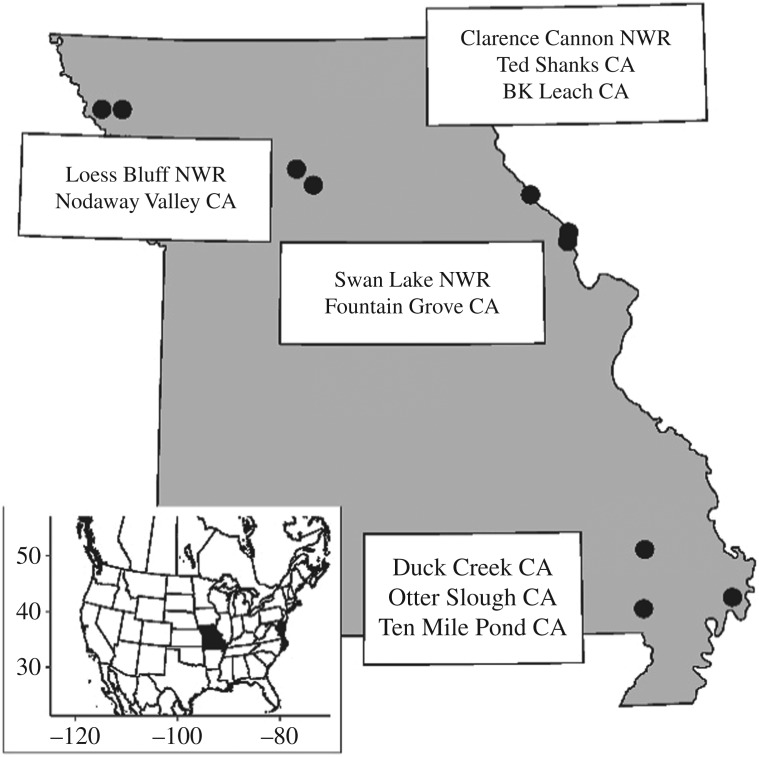
Study sites in Missouri, USA surveyed for sora during autumn migration 2012–2016. CA: Conservation Area, state land managed by the Missouri Department of Conservation; NWR: National Wildlife Refuge, federal land managed by the US Fish and Wildlife Service.

We selected public (i.e. MDC Conservation Areas and USFWS National Wildlife Refuges, figure 1), intensively managed wetland areas from across Missouri, from all wetlands within the state, because of their active moist soil management and historic importance for migrating waterfowl [15]. Areas were chosen (10 total) from wetland complexes in four regions (figure 1; electronic supplementary material, table S1). Regions are areas of two to four National Wildlife Refuges or Conservation Areas that are close to each other and share similar hydrology, surrounding landscape characteristics and weather. Wetland managers assigned to areas selected for surveys were consulted regarding the wetland impoundments under a moist soil management regime, and survey sites were selected from these impoundments. We surveyed moist soil wetland impoundments (a wetland surrounded by a levee, with manual water-level manipulation; 4.5–300 ha in size; mean = 26.5 ha; table 1) using selection criteria based on whether or not we were able to access the impoundment with an all-terrain vehicle (ATV) because some have interior ditches that cannot be crossed, and whether or not we could complete a survey within an hour and a half survey window.

**Table 1.** Survey start and end dates for each year of autumn surveys of sora (*Porzana carolina*) in Missouri, USA.

year	start date	end date	visits per state property	visits per federal property	number of impoundments	number of sora detected	number of available vegetation points	number of sora-used vegetation points
2012	17 August	7 October	3	3	40	1895	900	909
2013	11 August	27 October	3	4	39	1876	1890	624
2014	12 August	22 October	4	4	33	1268	2268	589
2015	12 August	23 October	4	4	33	1063	1710	522
2016	10 August	20 October	4	4	33	1803	2124	664

### 2.2. Surveys

We conducted surveys by driving systematic nocturnal transects spaced 30 m apart on ATVs for 3 h each night, starting a half hour after sunset [16]. We spaced these transects based on work by Fournier & Krementz [16] who equipped sora with very high frequency transmitters to examine the behaviour of sora in response to the survey method [16]. They determined that transects spaced 30 m apart were sufficient to avoid double counting [16]. Transects were completed in a serpentine fashion across an entire impoundment, so the length of transect varied with impoundment size [16]. We used a spotlight to scan for flushing, walking or swimming sora, while the observer was slowly moving along the transect on the ATV and took a global positioning system (GPS) location at the point where an individual was first detected. We surveyed in each of 5 years from August through to October and visited each National Wildlife Refuge or Conservation Area four times a year (roughly every two weeks; figure 1 and table 1; electronic supplementary material, table S1).

### 2.3. Vegetation data

We collected habitat data during daylight hours following a night of surveys under an available versus used framework. This approach is commonly carried out in the habitat use literature [17–20] and involves collecting the same kind of habitat data at available points and used points. Available points are defined as random points (randomly distributed within the boundary of the impoundment using the spsample() function in R, [21,22]) within the wetland impoundments surveyed which, together with the GPS points where sora were observed on surveys, allowed us to examine sora habitat use. Sora-used points (i.e. used points) are points where sora were detected, and collectively, these used points represent the habitat used by sora at the time of the survey. We sampled 20 random available points within each wetland impoundment each time the impoundment was surveyed for sora. We sampled vegetation associated with up to 20 sora points within each wetland impoundment each time the impoundment was surveyed; however, if habitat data were collected from less than 20 used points, it was because less than 20 sora were detected the previous night.

**Figure 2.**
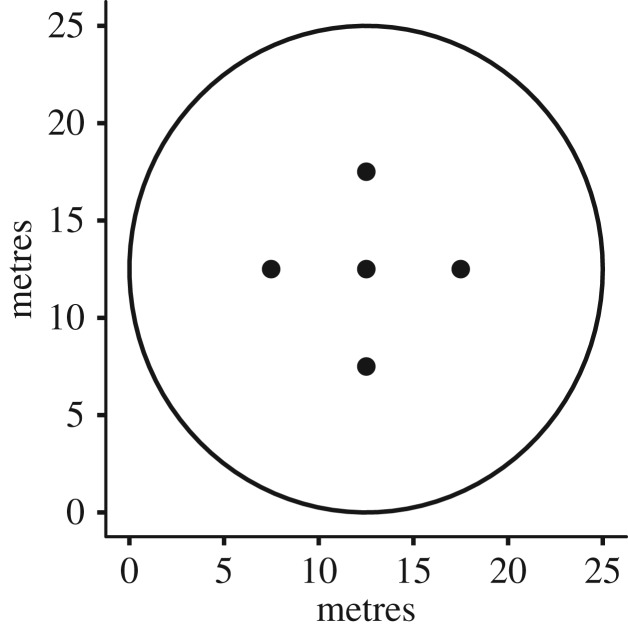
Each habitat plot was 25 m in diameter; the space within this 25 m diameter circle is the area over which the coverage of our vegetation variables was estimated. The five points are the five places where water depth was measured to the nearest centimetre, the centre of the plot, and 5 m away from the centre in each cardinal direction.

We placed 25 m diameter plots around each available and sora-used point and, because we were interested in habitat use by individual sora, we considered the plot (available and used) to be the experimental unit (figure 2). We measured water depth at (i) the centre of the plot and (ii) 5 m from the centre in the four cardinal directions (figure 2); we used the mean of these five measurements to characterize water depth. We visually estimated and created a sketch in the field to record the per cent cover of plant groups in each plot and placed them in the following categories: (i) annual moist soil plants, (ii) perennial moist soil plants, (iii) upland vegetation, (iv) woody vegetation, and (v) open areas [23,24].

Annual moist soil plants are those that fall below the water surface at the end of the growing season, including *Polygonum* spp. and *Echinochloa* spp. [2]. Perennial moist soil plants are those that stay above the water surface at the end of the growing season, including *Typha* spp. and *Sparganium* spp. Woody vegetation was predominantly *Salix* spp. and *Cephalanthus occidentalis*. Upland vegetation was composed of a wide suite of terrestrial annual plants, including upland grasses, *Solidago* spp., *Asclepias* spp. and *Helianthus* spp. Open areas are those without any vegetation and could be either bare earth or open water surface.

### 2.4. Analysis

We examined sora habitat use by comparing sora-used versus available habitat plots in a binomial mixed model framework. Our response variable was a binary of whether a point was used (1) or available (0). Our observations of sora among visits were not independent because of the sora's long stopover duration (40+ days, [25]). Owing to the lack of independence, we nested visit within year in a repeated-measures framework as a part of a binomial mixed model in the R package ‘lme4’ ([18,19], v. 1.1–12, R v. 3.3.2). We included mean water depth squared, and per cent cover of annual moist soil plants, perennial moist soil plants, woody vegetation, upland vegetation and open area, as fixed effects with random effects (random intercepts) of year. We included mean water depth squared because we expected habitat use to increase at some medium depth and decrease thereafter. We include a random effect of year because of the variation in weather and precipitation conditions among years. We considered a fixed-effect coefficient to be significant if the estimated coefficient and its 95% confidence interval did not overlap with 1. All data and code to reproduce these analyses are available in DataDryad [26].

## 3. Results

**Figure 3.**
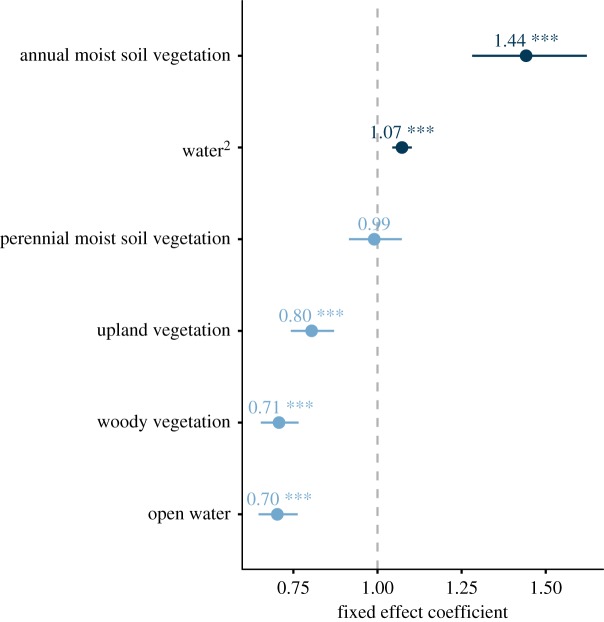
Fixed effects coefficients from binomial mixed models comparing sora (*Porzana carolina*) used versus available habitat from wetland impoundments surveyed during 2012–2016 in Missouri, USA. Line represents the 95% confidence interval around the point. *** indicates a significant difference because the 95% confidence interval does not overlap 1. Items to the left of the dotted line showed negative selection (selected less often than available); items to the right showed positive selection (selected more often than available).

We detected 7905 sora during August to October 2012–2016 (for the number of sora detected per year, table 1). We found significant positive use (sora using annual moist soil vegetation at higher percentages than is available; figure 3 and table 2) for annual moist soil vegetation and average water depth squared. On average, sora used water depths approximately 12 cm (yearly averages between 1.5 and 19.1 cm; electronic supplementary material, table S2); on average, sora used annual moist soil vegetation per cent cover of 37% (yearly averages ranging from 21 to 70%; electronic supplementary material, table S2), showing that they are positively using habitat with shallow water depths and annual plant cover.

**Table 2.** Model results of binomial mixed model comparing sora (*Porzana carolina*) used habitat points with available points from moist soil wetlands in Missouri, USA from 2012 to 2016.

covariate	estimate	standard error	*p*-value
intercept	−1.26	0.28	<0.001
open	−0.40	0.04	<0.001
upland	−0.30	0.04	<0.001
perennial	0.002	0.03	0.94
average water depth squared	0.05	0.01	<0.001
annual moist soil vegetation	0.28	0.05	<0.001
woody vegetation	−0.38	0.04	<0.001

random effects	variance	standard deviation	
visit nested within year	1.01	1.006	
year	0.01	0.113	

We found significant negative use (sora using lower percentages than available) with upland vegetation, woody vegetation and open areas (figure 3 and table 2; electronic supplementary material, table S2). We found no significant relationship with the rate at which sora used locations characterized as perennial moist soil vegetation (6.2% average sora used; electronic supplementary material, table S2).

## 4. Discussion

Sora are often considered to be a habitat generalist among the rails [8], and our findings support this portrayal. Although we found sora in a wide variety of water depths (dry to over 50 cm) that speaks to their adaptability, including their ability to swim and dive [27], most sora used shallow water depths when available. The median water depth used by sora was similar to the depth typically associated with the breeding season [28], deeper than previous autumn migration work [3,10–12] and overlaps with that used by migratory teal during autumn migration [3,29]. Other rails (Virginia rail, *Rallus limicola* and yellow rail, *Coturnicops noveboracensis*) use drier habitats during autumn migration [30].

Our work supports previous characterizations of sora autumn migration habitat, which showed selection for annual seed-producing plants, with seeds being their primary autumn food source, in combination with tall dense cover [10,31]. Sora diet diversifies in the spring and includes a higher proportion of aquatic invertebrates [8]; this shift is probably in preparation for the breeding season when proteins, lipids and minerals are needed for egg production [32,33] and other life-history events (i.e. molting). Similarly, a shift in sora habitat use occurs in spring as plant structure and cover for nesting and raising young become more important, whereas during autumn migration plants that provide the greatest energetic resources (i.e. seeds) are needed.

Many of the wetland impoundments we surveyed were largely dry during our first visits in early August. While sora are selecting deeper water when it is present, it is not widely available early in the autumn migration season. The limited area of inundated wetlands could impact the quality of autumn stopover habitat by affecting the ability of sora to obtain resources needed to continue migrating. Gries *et al*. [13] and Rundle & Fredrickson [11] also found that sora congregated around flooded areas early in autumn migration. Sora have long stopovers during autumn migration [25], often over 40 days, suggesting habitat quality during that stopover is very important. Frequently, the timing of managed moist soil wetland flooding is directed at meeting the needs of migratory waterfowl, which migrate later in the autumn, and hence may not meet the needs of sora because of the mismatch in timing [14,34].

The large-scale loss of palustrine wetlands makes science-based management of remaining wetlands of special concern. The timing of management actions is critical to provide suitable habitat when it is needed by multiple species, including rails, waterfowl, other waterbirds, reptiles and amphibians [11,35]. Here, we present a solid foundation to support future research and management of wetland habitats by understanding habitat use of one migratory wetland bird, which complements similar efforts for other species (i.e. waterfowl and shorebirds). This project was conducted at a broad scale among multiple areas and highlights a potential gap between sora habitat needs during autumn migration and timing of managed water-level manipulations. We suggest future research that is conducted at a finer resolution scale that can focus more closely on the within area and/or within impoundment resources made available by management actions. This combined with the information from this project will enable public land managers to more thoughtfully consider the trade-offs involved when considering multiple objectives aimed at multiple wetland-dependent species and various stakeholder groups.

## Supplementary Material

Supplementary Table I – Impoundments Surveyed from 2012-2016.

## Supplementary Material

Supplementary Table II. Summary of available habitat mean, minimum and maximum values across and by year for wetland impoundments in Missouri, USA surveyed for Sora (Porzana carolina) from 2012-2016

## Data accessibility

Our data and associated analysis scripts are publicly available here (http://dx.doi.org/10.5061/dryad.5n7t4) [26].
